# Subregional Differences in Alcohol Modulation of Central Amygdala Neurocircuitry

**DOI:** 10.3389/fnmol.2022.888345

**Published:** 2022-07-05

**Authors:** Mariam Melkumyan, Yuval Silberman

**Affiliations:** Department of Neural and Behavioral Sciences, Penn State University College of Medicine, Hershey, PA, United States

**Keywords:** lateral central amygdala, medial central amygdala, acute and chronic alcohol, glutamatergic transmission, GABAergic transmission

## Abstract

Alcohol use disorder is a highly significant medical condition characterized by an impaired ability to stop or control alcohol use, compulsive alcohol seeking behavior, and withdrawal symptoms in the absence of alcohol. Understanding how alcohol modulates neurocircuitry critical for long term and binge-like alcohol use, such as the central amygdala (CeA), may lead to the development of novel therapeutic strategies to treat alcohol use disorder. In clinical studies, reduction in the volume of the amygdala has been linked with susceptibility to relapse to alcohol use. Preclinical studies have shown the involvement of the CeA in the effects of alcohol use, with lesions of the amygdala showing a reduction in alcohol drinking, and manipulations of cells in the CeA altering alcohol drinking. A great deal of work has shown that acute alcohol, as well as chronic alcohol exposure via intake or dependence models, alters glutamatergic and GABAergic transmission in the CeA. The CeA, however, contains heterogeneous cell populations and distinct subregional differences in neurocircuit architecture which may influence the mechanism by which alcohol modulates CeA function overall. The current review aimed to parse out the differences in alcohol effects on the medial and lateral subregions of the CeA, and what role neuroinflammatory cells and markers, the endocannabinoid system, and the most commonly studied neuropeptide systems play in mediating these effects. A better understanding of alcohol effects on CeA subregional cell type and neurocircuit function may lead to development of more selective pharmacological interventions for alcohol use disorder.

## Central Amygdala and Alcohol Use Disorder

Alcohol use disorder (AUD) is a medical condition affecting around 15 million individuals in the US annually, characterized by an impaired ability to stop or control alcohol use despite adverse social, occupational, or health consequences (Understanding Alcohol Use Disorder, 2021). Long term alcohol use can result in dysfunction of various brain regions associated with the different symptoms of AUD: dysfunction of the prefrontal cortex is associated with Korsakoff’s syndrome, disinhibition, and impulsivity; atrophy of the cerebellum is associated with loss of coordination and low performance on executive function tests; damage to the hypothalamus and the limbic system, including the hippocampus, the bed nucleus of the stria terminalis, and the amygdala, has been shown to lead to amnesia and sensitized stress response (for a more detailed review please see Oscar-Berman and Marinković, 2007). For the purpose of this review, the discussion is focused on the central amygdala (CeA) as it is linked to the processing of motivational stimuli, anxiety, and negative affective states in alcohol use and withdrawal, critical aspects of continued alcohol use (Oscar-Berman and Marinković, 2007; Koob and Volkow, 2010; Roberto et al., 2020). For example, a fMRI study shows that individuals that have relapsed to AUD have a reduction in the volume of the amygdala compared to healthy controls (Wrase et al., 2008). Additionally, preclinical studies in male Sprague Dawley rats and C57Bl/6J mice have shown the involvement of the amygdala in alcohol intake, with lesions of the amygdala showing a reduction in voluntary alcohol drinking, and manipulations of cells in the CeA altering alcohol drinking (Möller et al., 1997; Roberto et al., 2020; Torruella-Suárez et al., 2020).

To understand the role of CeA neurocircuitry on alcohol drinking, studies have examined how acute and chronic alcohol alter glutamatergic and GABAergic transmission in the CeA (Silberman and Winder, 2015; Herman et al., 2016; Roberto et al., 2020). For the purpose of this review, acute alcohol exposure refers to a bath application of alcohol directly to CeA containing brain slices, while chronic alcohol use refers to long-term alcohol drinking or alcohol vapor exposure *in vivo*. As the CeA contains heterogeneous subregions, alcohol effects on CeA subregion neurocircuits are also heterogenous. For instance, some studies show an increase in glutamatergic transmission in the lateral subregion of the CeA following acute alcohol (Silberman et al., 2015; Melkumyan et al., 2022), other studies show a decrease in glutamatergic transmission in the medial CeA (Roberto, 2004; Herman et al., 2016). To better understand the role of the CeA in the development and maintenance of AUD, it is important to parse out the subregional differences on alcohol’s effects on CeA GABAergic and glutamatergic transmission. This review will provide an overview of studies on alcohol effects on CeA neurotransmission, with an emphasis on subregional differences and the potential roles these subregions may play in alcohol-related behaviors.

## Central Amygdala Circuitry

The CeA consists of the lateral (CeA_*L*_), medial (CeA_*M*_), and lateral capsular (CeA_*C*_) subdivisions (Allen Brain Atlas, 2021). The CeA receives inputs from the basolateral amygdala (BLA), the cortex, the thalamus, and other brain regions. It is thought that the majority of these glutamatergic inputs first interact with CeA_*L*_, with only some of these inputs directly projecting to the CeA_*M*_. The CeA_*L*_ and the CeA_*M*_ then communicate through mostly GABAergic circuits, with the CeA_*L*_ projecting to the CeA_*M*_, and the CeA_*M*_ projecting to other regions of the brain such as the hypothalamus, the locus coeruleus, the nucleus of the solitary tract, and others. (for further review please see Gilpin, 2012b; Babaev et al., 2018). In addition to projecting to the CeA_*M*_, the CeA_*L*_ also projects to other brain regions, such as the bed nucleus of the stria terminalis (BNST) (Gilpin, 2012b). These complex connections both within and outside of the CeA to brain regions involved in motivation and reward make the CeA an important region for functions such as emotional processing and addiction. However, our understanding of CeA neurocircuitry is currently in the process of being updated as new studies continue to inform on heterogenous populations of GABAergic neurons in the CeA that co-express neuromodulatory transmitters and peptides (Gilpin, 2012b; Sosulina et al., 2015; Kim et al., 2017; Hardaway et al., 2019; Beyeler and Dabrowska, 2020; Neugebauer et al., 2020; Liu et al., 2021; Borrego et al., 2022; Douceau et al., 2022). Although a complete assessment of CeA neurocircuit heterogeneity and microcircuit function is out of the scope of this review, some of the most relevant findings from this rich literature as it relates to alcohol modulation of CeA circuit function is discussed in “Modulators of Proposed Circuit” Section of this review.

## Overview of Proposed Circuit Mechanism in Relation to Alcohol Studies

**FIGURE 1:**
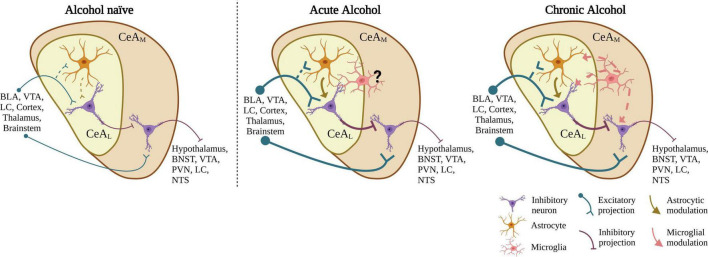
Proposed CeA neurocircuit model and summary alcohol effects on CeA neurotransmission. In alcohol-naive circuitry, the CeA_*L*_ receives glutamatergic input from various brain regions and sends GABAergic projections to the CeA_*M*_. The CeA_*M*_, which also receives some direct input from similar regions as the CeA_*L*_, in turn sends GABAergic projections to various brain regions. Acute application of EtOH to CeA slices increases glutamatergic neurotransmission in the CeA_*L*_, an effect regulated by astrocytic function. We propose the increased glutamatergic release acts on CeA_*L*_ neurons leading to an increase in GABA release in the CeA_*M*_. After chronic alcohol exposure, in addition to astrocytic function, microglia may also play a role in the increase in glutamatergic transmission through upregulation of activated microglial activity. BLA, basolateral amygdala; VTA, ventral tegmental area; LC, locus coeruleus; BNST, bed nucleus of the stria terminalis; PVN, paraventricular nucleus; NTS, nucleus of the tractus solitarius. Created with BioRender.com.

Most neurons in the CeA are GABAergic, including both GABAergic projection neurons and local interneurons. Overall, studies show that acute alcohol application increases GABAergic transmission in the CeA_*M*_, decreases glutamatergic transmission in the CeA_*M*_, and increases spontaneous glutamatergic transmission in the CeA_*L*_ (see further sections below, Roberto, 2004; Silberman et al., 2015; Roberto et al., 2020; Melkumyan et al., 2022). The concentration of acute bath alcohol application has been extensively studied through concentration response curves in alcohol naïve rodents (see Roberto et al., 2004; Silberman et al., 2015) showing the apparent IC50 at physiologically relevant concentrations of roughly 20 mM (∼0.092% blood ethanol concentration) for both glutamatergic neurotransmission in mice (Silberman et al., 2015) and GABAergic neurotransmission in rats (Roberto et al., 2004). In both cases, acute alcohol reaches a ceiling effect after ∼50 mM. Since CeA_*L*_ provides direct GABAergic projections to the CeA_*M*_, it is proposed that alcohol-induced increase in glutamatergic signaling in the CeA_*L*_ may directly lead to the well-established alcohol-enhancement of GABA release in the CeA_*M*_. Recent findings indicate the acute alcohol-induced increase in glutamatergic transmission in the CeA_*L*_ is mediated by astrocytes, with potential microglial mediation (Melkumyan et al., 2022), and additional findings indicate that microglia may be critical regulators of CeA GABA transmission in chronic alcohol exposure models (Warden et al., 2020), suggesting neuroimmune signaling in general may be a critical component of alcohol action in the CeA. The effect of alcohol on GABAergic transmission in the CeA_*L*_ is currently unknown. These findings that led us to develop a simplified circuit design (Figure 1). How this circuit is modulated by alcohol and select transmitter/peptide systems is reviewed in the followed sections. This simplified circuit will need to evolve based on recent and likely future research on alcohol effects on heterogenous neuronal subtypes which have subregional microcircuit selectivity in the CeA subregions.

## GABAergic Transmission

### Medial Central Amygdala

Alcohol enhances GABA signaling both presynaptically and postsynaptically in CeA_*M*_ slices in both alcohol naïve and alcohol dependent rodents (Roberto et al., 2003, 2004; Agoglia and Herman, 2018). In chronic intermittent alcohol vapor treated Sprague Dawley rats, there was an increase in GABAergic transmission at baseline compared to naïve rats 2–8 h after the last vapor exposure (Roberto et al., 2004). Additionally, in the same chronic alcohol treated rats, there was an increase in the probability of GABA release compared to naïve rats, suggesting that chronic alcohol enhances alcohol-induced release of GABA (Roberto et al., 2004). After an acute bath application of 44 mM alcohol, there was a significant increase in action-potential dependent presynaptic GABAergic transmission in both naïve and chronic alcohol vapor treated Sprague Dawley rats and C57Bl/6J mice (Roberto et al., 2004; Harris et al., 2017; Varodayan et al., 2017b; Bajo et al., 2019). This is noteworthy, as despite the increased GABAergic transmission in basal conditions, bath application of alcohol led to an equal increase in GABAergic transmission between naïve and alcohol treated rats, suggesting a lack of tolerance to the effect of acute alcohol (Roberto et al., 2004; Harris et al., 2017; Varodayan et al., 2017b; Tunstall et al., 2019; Khom et al., 2020). Interestingly, when looking at action-potential independent GABAergic transmission, the effect of acute 44 mM alcohol application seems to be higher in rats and mice exposed to chronic alcohol compared to naïve rodents (Roberto et al., 2004; Harris et al., 2017). Additionally, 44 mM alcohol also increased postsynaptic GABAergic transmission in some cells in the CeA_*M*_ (Roberto et al., 2004), suggesting that even though primary effects of alcohol on CeA_*M*_ circuits are presynaptic, there may also be some postsynaptic effects. These studies suggest the effect of acute bath application of alcohol on action potential-independent GABAergic transmission in the CeA_*M*_ is sensitized by alcohol dependence. However, the effect of acute alcohol on action potential-dependent GABAergic mechanism is not altered after alcohol dependence. It is worth noting that the effect of alcohol on GABAergic transmission in the CeA_*M*_ may differ between strains. In naïve rats, msP, Sprague Dawley, and Wistar rats had an increase in presynaptic spontaneous action potential-independent GABAergic activity compared baseline (Herman et al., 2013). However, only Sprague Dawley and Wistar rats had an increase in postsynaptic spontaneous action-potential independent activity (Herman et al., 2013). Therefore, the use of different rat strains can uncover some of the postsynaptic effects of alcohol. Additionally, chronic alcohol has been shown to alter noradrenergic sensitivity of CeA GABAergic transmission but acute alcohol effects on CeA GABAergic transmission are not regulated by noradrenergic signaling (Varodayan et al., 2022).

### Lateral Central Amygdala

While the GABAergic transmission has been extensively studied in the CeA_*M*_, to our knowledge there have been no studies looking at alcohol effects on GABAergic transmission in the CeA_*L*_. It is worth noting that many studies do not specify the subregion of the CeA being studied (see Table 1), however, as most of these studies were performed in the same lab, these findings were likely in the CeA_*M*_. Therefore, it is worth exploring how alcohol affects the GABAergic transmission in the CeA_*L*_, compared to the CeA_*M*_.

**TABLE 1:** Summary of the data from multiple papers looking at GABAergic transmission in rats and mice through evoked (eIPSC), spontaneous (sIPSC), and mini (mIPSC) inhibitory postsynaptic currents (IPSC).

Paper	Rodent type	Sex	CeA subregion	Method	Changes in GABAergic transmission
Agoglia et al., 2022	CRF1:GFP mice	Male and female	Medial	sIPSC	No changes in basal activity after voluntary alcohol drinking in CRFR1 neurons In control males, decrease in sIPSC frequency after 44 mM alcohol exposure in CRFR1 neurons
					In voluntary alcohol drinking males, increase in sIPSC frequency after 44 mM alcohol exposure in CRFR1 neurons
					In males (both control and alcohol drinking), no differences in sIPSC amplitude in CRFR1 neurons
					In females (both control and alcohol drinking), no differences in sIPSC frequency or amplitude
					Voluntary alcohol drinking sensitized sIPSC frequency in males and females in CRFR1 neurons
Kirson et al., 2021	Sprague Dawley Rats	Male and female	Medial	sIPSC	In males, increase in frequency after alcohol dependence Females have faster kinetics than males as measured by rise and decay time
					Naïve rats have faster decay time than dependent rats
					Increase in frequency in males but not in females after 44 mM alcohol application
					Increase in frequency in dependent females after a high dose of alcohol (88 mM)
					Increase in frequency in naïve and dependent males after 44 and 66 mM alcohol
					Increase in frequency in naïve but not dependent male after 88 mM alcohol
Bloodgood et al., 2021	PdynIRES-Cre × Gt(ROSA26)SorloxSTOPlox-L10-GFP, mice	Male and female	Medial and lateral	sIPSC	No changes in baseline sIPSC frequency, amplitude, or kinetics after alcohol drinking through 3 sessions of drinking in the dark paradigm No differences in males and females
Agoglia et al., 2020	CRF1:GFP mice	Male and female	Medial	sIPSC	No baseline differences in sIPSC between males and females Decreased frequency after 44 mM alcohol application in CRFR1+ neurons in males but not females
					No changes in sIPSC amplitude in males and females
Khom et al., 2020	Sprague Dawley rats	Male	Medial	sIPSC	Increased sIPSC frequency in naïve and dependent rats after 44 mM alcohol – effect was sensitized with substance P application
					Increased rise and decay time in naïve rats after 44 mM alcohol
Warden et al., 2020	C57Bl/6J mice	Male	Medial	sIPSC	No differences in basal frequency, amplitude, or kinetics in dependent and naïve mice Microglial depletion reduced frequency, amplitude, and decay time in naïve and dependent mice
Bajo et al., 2019	C57Bl/6J mice	Male	Medial	sIPSC	Increased frequency, rise time, and decay time after 44 and 100 mM alcohol compared to baseline
Patel et al., 2019	C57Bl/6J mice	Male	Medial	sIPSC	Increased frequency after 44 mM alcohol application in naïve, alcohol exposed, and alcohol dependent mice
					IL-1B had no effect on acute alcohol effect
Tunstall et al., 2019	Sprague Dawley rats	Male	Medial	eIPSC	Increased amplitude in dependent and non-dependent rats after 44 mM alcohol application
				sIPSC	Increased frequency in dependent and non-dependent rats after 44 mM alcohol application
Varodayan et al., 2017b	Sprague Dawley rats	Male	Medial	sIPSC	Increased basal frequency in alcohol dependent rats compared to naïve Increased frequency in naïve and dependent rats after 44 mM alcohol
Harris et al., 2017	Wistar Rats	Male	Medial	sIPSC	Increased frequency in dependent wild type, but not TLR4 knockout, rats after 44 mM alcohol exposure
					Decreased frequency after 44 mM alcohol application in LPS injected wild type but not TLR4 knockout rats
				mIPSC	Increased frequency in non-dependent wild type and TLR4 knockout rats after 44 mM alcohol
Schweitzer et al. (2016)	Alk KO mice (Alk−/−) or C57Bl/6J mice (Alk+/+)	Male	Medial	sIPSC	Increased frequency after alcohol application in Alk+/+ and Alk−/− mice with a more pronounced effect in Alk−/− mice
					No sensitization after acute alcohol in any of the groups
Bajo et al., 2015	B6129SF2/J mice	Male	Medial	eIPSC	Increased amplitude after 44 mM alcohol application
				mIPSC	Increased frequency, rise time, decay time after 44 mM alcohol application
					Alcohol effect on frequency reduced in some but not other cells after IL-1B application
Gilpin et al., 2011	Sprague Dawley rats	Male	Unknown	eIPSP	Increase in IPSP amplitude (reversed by NPY application)
Bajo et al., 2008	PKCe+/+ and	Male	Unknown	eIPSP	Increased amplitude and increased GABA release through decreased PPF after 44 mM alcohol exposure in PKCe+/+ but not in PKCe−/− mice
	PKCe−/− mice			mIPSC	Increased frequency but not amplitude after 44 mM alcohol application in in PKCe+/+ mice
					Decreased frequency but not amplitude after 44 mM alcohol application in PKCe−/− mice
Nie et al., 2004	C57Bl/6J mice	Unknown	Unknown	eIPSC	Increased IPSC amplitude in wild type but not CRF1 KO mice after 44 mM alcohol application Increased probability of release after 44 mM alcohol
					Alcohol effect blocked by CRF1/2 antagonist but not CRF2 antagonist
Roberto et al., 2004	Sprague Dawley rats	Male	Medial	eIPSP/C	Increased IPSP/IPSC amplitude in chronic alcohol treated and naïve rats
				mIPSC	Frequency and amplitude higher in chronic alcohol treated rats compared to naïve
					Frequency increased compared to baseline in naïve and chronic alcohol treated rats
Roberto et al., 2003	Sprague Dawley rats	Male	Unknown	eIPSP/C	Increased isolated GABA mediated IPSC amplitude after 44 mM alcohol Increased GABA release after 44 or 66 mM alcohol of GABA IPSP/C
				sIPSC	Increased frequency after 44 mM alcohol
				mIPSC	Increased frequency and amplitude after 44 mM alcohol

### Sex Differences

Most studies examining GABAergic transmission in the CeA so far have predominantly focused on male rodents (see Table 1). However, a recent study found potential sex differences in the response of spontaneous inhibitory neurotransmission to acute alcohol in naïve and alcohol dependent rats (Kirson et al., 2021). Specifically, in male rats, 44–88 mM bath alcohol application significantly increased the frequency of inhibitory spontaneous events in the CeA_*M*_ compared to baseline, a result seen in previous studies. This increase in presynaptic inhibitory activity was not seen in naïve or alcohol dependent female rats at 44 mM concentration of alcohol, regardless of the estrous cycle stage. At a higher (88 mM) concentration of alcohol, there was a significant increase in the spontaneous inhibitory event frequency compared to baseline in dependent, but not naïve, females. This suggests that acute alcohol application does not have any effect on inhibitory transmission at any concentration in alcohol naïve females and that chronic alcohol exposure induces mild sensitivity to the effects of acute alcohol (Kirson et al., 2021). It is worth noting that initial studies examining concentration response curves of acute bath application of alcohol were done only in males (Roberto, 2004; Silberman et al., 2015), while the finding by Kirson et al. (2021) was in females. Whether sex as a biological variable is critical for acute alcohol effects on CeA neurotransmission in naïve and dependent animals across species is still an open question, but our recent work (Melkumyan et al., 2022) indicates that, at least for spontaneous glutamatergic transmission in the CeA_*L*_, the magnitude of acute alcohol induced effects is similar in alcohol-naïve male and female mice. The Kirson et al. (2021) study showed significant differences in the kinetics of GABAergic transmission, with naïve and alcohol dependent females having a faster rise and decay times than males, indicating sex differences in the postsynaptic GABA_*A*_ receptor activity after alcohol exposure. The effect was dependent on the estrous cycle with proestrus and metestrus/diestrus females having faster kinetics compared to estrus females.

Another study by Agoglia et al. (2020) showed differential effect of 44 mM alcohol on corticotropin releasing factor 1 receptor (CRF1R+) neurons in the CeA_*M*_ with alcohol-naïve CRF1:GFP male mice showing a significant decrease in spontaneous inhibitory presynaptic activity compared to baseline, while females showed only a trend toward reduction in spontaneous activity. The study did not find any statistically significant sex differences but suggested that acute alcohol reduces GABA release onto CRF1R+ neurons in males and not in females (Agoglia et al., 2020). The same group followed up on these experiments and saw that voluntary alcohol drinking through the two-bottle choice paradigm led to an increase in presynaptic spontaneous GABAergic transmission in CRF1R+ neurons in the CeA in males only, with no changes in females and no sex differences in postsynaptic activity (Agoglia et al., 2022). It is worth noting that there were no sex or treatment group (water or alcohol) differences in baseline GABA activity. Similarly, a study by Bloodgood et al. (2021) saw no sex differences in synaptic spontaneous GABAergic transmission after three cycles of drinking in the dark paradigm in preprodynorphin (PDYN) neurons in the CeA (subregion not described). However, there were sex-specific differences in the intrinsic excitability of PDYN neurons following the drinking in the dark paradigm, which may be related to subtle sex-differences noted in drinking behaviors in this model (Bloodgood et al., 2021).

In general, the studies in the CeA_*M*_ show that alcohol increases GABAergic transmission, at least in males. Chronic alcohol exposure sensitizes the effect of subsequent acute alcohol on action potential dependent spontaneous GABAergic transmission in male rats with a smaller sensitization effect in female rats that may be dependent on estrous cycle.

### Additional Considerations for Alcohol Effects on Central Amygdala GABA Signaling

GABA acts on two types of receptors, the ionotropic ligand gated GABA_*A*_ receptor and the metabotropic G-protein coupled GABA_*B*_ receptor. Synaptic GABA_*A*_ receptors mediate fast or phasic inhibition, peri- and extrasynaptic GABA_*A*_ receptors can mediate tonic inhibition, and GABA_*B*_ receptors mediate slow synaptic inhibition postsynaptically and regulate GABA release presynaptically. Alcohol has been shown to act on both the GABA_*A*_ and GABA_*B*_ receptors (Roberto et al., 2003; Elvig et al., 2021), although it is still unclear if alcohol can directly modulate GABA_*A*_ or GABA_*B*_ function via actions at specific binding sites or if alcohol effects on these receptors occurs via indirect mechanisms.

The effect of alcohol on GABA_*A*_ receptors is more robust than the effects of GABA_*B*_, with alcohol exposure affecting the cycling of synaptic and extrasynaptic GABA_*A*_ receptors and the various subunits of the receptor (Liang and Olsen, 2014). Synaptic GABA_*A*_ receptors are most commonly composed of two α, two β, and one γ subunits, while peri- and extrasynaptic receptors typically use δ subunits instead of γ (Liang and Olsen, 2014). Alcohol can cause differential changes in protein and mRNA expression of various subunits of the GABA_*A*_ receptors (for detailed reviews see Lobo and Harris, 2008; Liang and Olsen, 2014; Stephens et al., 2017; Barker and Hines, 2020). For example, various studies have shown that specific combinations of subunits of the GABA_*A*_ receptors are more sensitive to alcohol than others (Lobo and Harris, 2008). Studies in humans with AUD found increased mRNA expression in the expression of α1, α4, α5, β1, and γ1 subunits in the hippocampal dentate gyrus (Jin et al., 2011). Additionally, studies from the same group have shown a significant decrease in mRNA encoding of the α2 subunit in the CeA of individuals with AUD (Jin et al., 2014).

Consistent with findings in humans, studies in rodents show changes in GABA_*A*_ receptor subunits after alcohol administration. Regardless of direct or indirect alcohol actions on GABA receptors themselves, targeting specific GABA_*A*_ receptor subunits in the CeA can alter alcohol intake and tolerance in rodent models (Liu et al., 2011; Elvig et al., 2021). Liu et al. (2011) used alcohol preferring P rats, which have elevated levels of α1 and α2 subunits, in a drinking-in-the-dark-multiple-scheduled-access protocol to induce binge drinking. Using siRNA to inhibit α2 expression in specific brain regions, it was found that α2, and not α1, overexpression in the CeA promotes alcohol drinking (Liu et al., 2011). Such α2 inhibition did not alter drinking when restricted to the ventral pallidum or the nucleus accumbens suggesting a regional specificity to the CeA. Studies have shown that the CeA predominantly has α1, α2, and α3-subunit containing GABA_*A*_ receptors, making the antagonists of these receptors an important tool for studying the effect of alcohol on GABAergic transmission through GABA_*A*_ receptors (Foster et al., 2004). Selective antagonism of α1 containing GABA_*A*_ receptors directly in the CeA resulted in a reduction of alcohol responding in an operant chamber, suggesting that α1 subunit containing GABA_*A*_ receptors in the CeA are involved in alcohol drinking behavior (Foster et al., 2004). The effect of GABA_*A*_ receptors on alcohol drinking behaviors seems to be associated with the alcohol effect on GABAergic transmission in the CeA.

Administration of direct GABA_*B*_ agonists, such as baclofen, or positive allosteric modulators (PAM) of GABA_*B*_ receptors suppresses alcohol related behaviors in rodents (Maccioni and Colombo, 2009; Agabio and Colombo, 2014; Elvig et al., 2021) such as: alcohol drinking in the intermittent alcohol access paradigm (Minnaard et al., 2021), acquisition of alcohol drinking in the two-bottle choice test (Colombo et al., 2002), binge drinking in the drinking in the dark (Hwa et al., 2014; Crabbe et al., 2017) and scheduled high alcohol consumptions experimental paradigms (Tanchuck et al., 2011), and alcohol self-administration in a dose-dependent manner (Anstrom et al., 2003; Liang et al., 2006). GABA_*B*_ receptor-induced suppression of alcohol related behaviors suggests that GABA_*B*_ receptors may be a target for reduction of alcohol drinking in clinical studies.

As described above, in the CeA specifically, alcohol has been shown to enhance GABAergic transmission at pre- and post-synaptic sites (Roberto et al., 2003). However, even though global GABA_*B*_ receptor activation leads to a decrease in alcohol drinking and motivation, GABA_*B*_ receptor agonists do not affect the alcohol induced amplitude increase of IPSPs and IPSCs in the CeA, suggesting that postsynaptic alcohol actions on GABA_*A*_ receptors are independent of GABA_*B*_ receptor activity (Roberto et al., 2003). However, it is unclear if alcohol increases in GABA release drive changes in GABA_*A*_ receptor function or if alcohol directly impacts these receptors.

## Glutamatergic Transmission

Although the vast majority of neurons in the CeA are GABAergic, local glutamatergic transmission has been implicated in acute and chronic effects of alcohol (Silberman et al., 2015; Kirson et al., 2018; Roberto et al., 2020; Elvig et al., 2021; Melkumyan et al., 2022). Glutamatergic signaling occurs via activation of ionotropic *N*-methyl-D-aspartic acid (NMDA) receptors, α-amino-3-hydroxy-5-methyl-4-isoxazolepropionic acid (AMPA) receptors, and metabotropic G protein coupled receptors (mGluRs). Studies have shown that acute alcohol reduces glutamatergic transmission in the CeA_*M*_ through NMDAR and non-NMDAR mediated mechanisms by inhibiting glutamate release (Roberto, 2004; Roberto et al., 2020). Conversely, chronic alcohol or alcohol-dependence sensitizes NMDARs to alcohol and up-regulates metabotropic glutamate receptor signaling (Roberto, 2004; Kufahl et al., 2011; Roberto et al., 2020). Most studies looking at alcohol effects on glutamatergic transmission in the CeA observe a reduction in the transmission (Gilpin and Roberto, 2012; Herman et al., 2016; Varodayan et al., 2017a; Kirson et al., 2018), unlike the potentiated effect of alcohol on GABAergic transmission (Table 2). However, a number of studies observed an increase in glutamatergic transmission after alcohol exposure (Silberman and Winder, 2015; Silberman et al., 2015; Melkumyan et al., 2022). The contradictory findings seem to be due the exposure length of alcohol (acute or chronic), mode of electrophysiologic recording, and the subregion of the CeA. Additionally, diet composition may play an important role in regulating alcohol modulation of CeA neurotransmission (Coker et al., 2020; Mahajan et al., 2021).

**TABLE 2:** Summary of the data from multiple papers looking at glutamatergic transmission in rats and mice through evoked (eEPSC), spontaneous (sEPSC), and mini (mEPSC) inhibitory postsynaptic currents (EPSC).

Paper	Rodent type	Sex	CeA subregion	Method	Changes in glutamatergic transmission
Melkumyan et al., 2022	C57Bl/6J mice	Male and Female	Lateral	sEPSC	Increased frequency after 20 mM alcohol application with no effect on amplitude. Alcohol effect on frequency attenuated after astrocyte inhibition
Warden et al., 2020	C57Bl/6J mice	Male	Medial	sEPSC	Decreased sEPSC frequency in alcohol dependent mice after microglia depletion Decreased rise and decay time after microglia depletion in both alcohol dependent and non-dependent mice
Bloodgood et al., 2021	PdynIRES-Cre × Gt (ROSA26)SorloxSTOPlox-L10-GFP, mice	Male and female	Unknown	sEPSC	No changes in sEPSC frequency, amplitude, or kinetics after alcohol drinking through 3 sessions of drinking in the dark paradigm No differences in males and females
				Current clamp	Increased action potential firing in male mice exposed to alcohol with no differences in resting membrane potential, action-potential threshold, or rheobase of PDYN neurons
					Slight reduction in action potential firing in female mice exposed to alcohol with no differences in resting membrane potential, action-potential threshold, or rheobase of PDYN neurons
Varodayan et al., 2017a	Sprague Dawley rats	Male	Medial	eEPSP	No differences between the PPF of naïve and dependent rats Reduced EPSP amplitude after CRF exposure in alcohol naïve and dependent rats
					Decreased glutamate release at CeA synapses in naïve and alcohol dependent rats after CRF
				mEPSC	Reduced frequency and amplitude in alcohol dependent rats compared to naïve rats
					Increased frequency in alcohol dependent and naïve rats after CRF application
					Decreased frequency after CRFR1 antagonist application in naïve rats
					Increased frequency after CRFR2 antagonist application in naïve rats
Kirson et al., 2018	msP and Wistar rats	Male and female	Medial	eEPSP	Increased baseline PPR in msP rats compared to Wistar rats Decreased amplitude after 44 mM alcohol exposure in Wistar male and female rats, and msP males, but not females
					Decreased amplitude after CB1R agonist in msP males and females, Wistar males, but not Wistar females with significant difference between msP and Wistar strains.
					CB1R agonist blocked effect of alcohol in Wistar females, induced an effect in msP females
					No changes in amplitude and no alterations of alcohol effect after CB1R antagonist
Logrip et al., 2017	Wistar rats	Male and female	Medial and lateral	eEPSP	No baseline differences in stimulus-response properties between sex or subdivision, with some modulation of presynaptic release in CeA_M_ by estrous cycle
					Decreased BLA evoked EPSP in males and females after 44 mM alcohol exposure in CeAL and CeA_M_
					No differences between the PPF in control or 44 mM alcohol treated cells in males and females in the CeA_L_ and the CeA_M_
					In CeA_L_ alcohol induced effects on EPSP were not different across estrous cycle
					In CeA_M_ alcohol increased eEPSP in proestrus but decreased in estrus cycle.
					Facilitation of interstimulus interval by alcohol during the estrus cycle for the 50 ms interstimulus interval with no changes in males in CeAM only
Herman et al., 2016	msP and Wistar rats	Male	Medial	sEPSC	Increased baseline frequency in msP rats compared to Wistar rats Decrease in frequency after 44 mM alcohol in some cells, increase in other cells
				mEPSC	No differences between baseline frequency in msP and Wistar rats
					Decrease in frequency after 44 mM alcohol in some cells, increase in other cells
				eEPSP	Elevated baseline amplitude in msP compared to Wistar rats
					Lower PPF in msP compared to Wistar rats suggesting increased glutamate release in msP rats
					Decreased amplitude after 44 mM alcohol
Silberman et al., 2015	C57Bl/6J mice	Male	Lateral	sEPSC	Increase in frequency, decrease in rise and decay time after 100 mM alcohol Effect of alcohol attenuated after CRFR1 and CRFR2 antagonist application
				Current Clamp	No changes in CRF neuron membrane potentials, decreased action potential amplitude after 100 mM alcohol
Kallupi et al., 2014b	Wistar rats	Male	Medial	eEPSP	Decreased amplitude after 44 mM alcohol exposure with no effect on PPR No differences in amplitude between naïve and alcohol dependent rats Decreased amplitude and PPR in alcohol dependent rats after acute alcohol exposure
Zhu et al., 2007	Wistar rats	Male	Medial	eEPSP	Reduced amplitude of non-NMDAR and NMDAR mediated EPSP after 44 mM alcohol Increased PPR in non-NMDAR and NMDAR mediated EPSP after 44 mM alcohol
					Reduced PPR after chronic alcohol treatment through NMDAR and non-NMDAR mechanism
				mEPSC	Reduced frequency and amplitude after 44 mM alcohol
Roberto et al., 2004	Sprague Dawley rats	Male	Medial	eEPSP/C	Reduced EPSP/C amplitude through non-NMDAR-mediated mechanism after 44 mM alcohol Decreased amplitude of evoked NMDAR-mediated EPSP/C after 44 mM alcohol
					Reduced non-NMDA transmission baseline after chronic alcohol exposure but no changes in acute alcohol effects compared to naïve
					Reduced amplitude of NMDAR-mediated EPSP in chronic alcohol exposed rats through sensitization to acute alcohol effect mediated by NR2B NMDAR subunits Decreased PPF of NMDAR-mediated EPSP/C in chronic alcohol exposed rats (increased glutamate release in chronic alcohol exposed rats)
					Sensitization of acute alcohol effect after chronic alcohol on postsynaptic level

### Medial Central Amygdala

Studies on the effects of alcohol on CeA_*M*_ have been mixed. Interestingly, similar to the effect on GABAergic transmission, the type of electrophysiological recording plays a role in uncovering the effects of alcohol on glutamate transmission. Most studies in the CeA_*M*_ show a reduction in glutamate transmission by alcohol (Gilpin and Roberto, 2012; Herman et al., 2016; Varodayan et al., 2017a; Kirson et al., 2018). However, rodent strain can play a significant role in glutamatergic transmission in the CeA_*M*_ and its modulation by alcohol. Marchigian Sardinian Preferring (msP) rats – a strain of rats selected for their high alcohol preference – have a higher basal frequency of spontaneous excitatory postsynaptic currents compared to Wistar control rats (Herman et al., 2016), suggesting heightened presynaptic glutamatergic release mechanisms in msP rats. Such baseline differences in action potential dependent presynaptic glutamatergic release between the msP and Wistar were not seen in action potential-independent recordings (Herman et al., 2016). Additionally, after 44 mM alcohol exposure, a subgroup of CeA neurons in both Wistar and msP rats had a significant increase in action potential-dependent spontaneous excitatory current frequency compared to baseline, and there were significant differences in the magnitude of this increase between the two strains. These strain differences were not seen in action potential-independent excitatory currents. It is worth noting, that a subgroup of CeA neurons in both Wistar and msP rats had a decrease in presynaptic action potential-dependent and independent excitatory currents. These results suggest that the difference between the strains may be due to action potential-dependent, and not independent, glutamatergic release mechanisms and potentially the cell-type being recorded. This result was further confirmed by looking at evoked glutamatergic activity, which showed that msP rats have increased probability of glutamate release based on paired pulse facilitation recordings (Herman et al., 2016). Kirson et al. (2018) also found strain differences, but instead of an increase in glutamate release, showed a decrease in evoked glutamate activity in msP rats compared to Wistar rats in baseline conditions. Both Herman et al. (2016) and Kirson et al. (2018) found that alcohol reduced the magnitude of evoked glutamatergic activity in both msP and Wistar rats, with no changes in glutamate release probability. These studies highlight the importance of electrophysiologic method chosen to study the effects of alcohol on glutamatergic activity in the CeA_*M*_.

It is unclear as to why these two studies saw differences in their findings during basal conditions with no differences during alcohol application. It is worth noting that Herman et al. (2016) only used males in their study, while Kirson et al. (2018) used both males and females, although the results found in Kirson et al. (2018) were consistent across males and females (see “Sex Differences” Section below). These studies suggest that further experiments are needed to understand the strain and recording type specific differences in alcohol effect on CeA_*M*_ glutamatergic transmission.

In addition to both strain differences and recording type, the duration of alcohol exposure plays a role in the effect of alcohol on glutamatergic transmission in the CeA. Acute alcohol has been shown to reduce glutamatergic activity through both an NMDAR and non-NMDAR mediated mechanism (Roberto, 2004; Roberto et al., 2020). Chronic alcohol vapor exposure has been shown to increase glutamate release at baseline conditions, however, 44 mM alcohol superfusion elicited a significantly greater reduction of glutamatergic transmission in chronic alcohol exposed rats compared to naïve rats, suggesting that chronic alcohol exposure sensitizes the NMDA receptors to subsequent alcohol effects (Roberto, 2004). While Roberto et al. (2004) saw a differential effect of acute vs. chronic alcohol, a study by Varodayan et al. (2017a) did not find any differences in the glutamate release of naïve and alcohol dependent rats. However, there were differences between alcohol dependent and naïve rats in spontaneous action-potential independent glutamatergic activity, with alcohol dependent rats having a reduced frequency and amplitude compared to naïve rats, suggesting that the effects of acute alcohol might be uncovered in action potential-independent mechanisms (Varodayan et al., 2017a). Although Roberto and Varodayan did not find any differences in naïve and alcohol dependent rats, Kallupi et al., 2014b showed that after acute alcohol exposure there was a reduction in glutamatergic transmission. After chronic alcohol exposure, when 44 mM alcohol was bath-applied, there was an increase in glutamate release, suggesting that chronic alcohol alters the response to acute alcohol through a presynaptic mechanism. Lastly, a study by Zhu et al. (2007) showed reduced glutamate release after 44 mM alcohol in non-dependent Wistar rats mediated by non-NMDAR and NMDAR mechanisms, but an increased release after chronic alcohol treatment, suggesting a reversal and alteration of the effect of acute alcohol after chronic alcohol treatment. Overall, the studies suggest that the actions of alcohol on the CeA_*M*_ glutamatergic transmission are (1) mediated by both presynaptic and postsynaptic mechanisms, (2) mediated by action potential dependent and independent mechanisms, (3) are strain specific, and (4) are dependent on the history of alcohol exposure (acute vs. chronic). The complexity of the mechanisms of action of alcohol speaks to the need of further studying the effect of alcohol on the CeA_*M*_ through a variety of techniques to parse out the exact points of alcohol action.

### Lateral Central Amygdala

The effect of alcohol on the glutamatergic transmission in the CeA_*L*_ is less extensively examined. A study by Silberman et al. (2015) explored the effect of alcohol on the CeA_*L*_ in alcohol naive male C57Bl6/J mice and saw an increase in presynaptic glutamatergic activity after acute alcohol application (5–100 mM) with an estimated IC50 of roughly 20 mM. Expanding on these findings, a recent study from our lab showed that acute bath application of 20 mM alcohol increased spontaneous action potential dependent glutamatergic signaling presynaptically in both male and female C57Bl/6J mice, with no sex differences found (Melkumyan et al., 2022). Additionally, the ability of acute alcohol to increase glutamatergic transmission was attenuated when astrocytes were inhibited either pharmacologically or chemogenetically, suggesting a critical role for astrocytes in the effect of alcohol on CeA_*L*_ glutamatergic transmission (Melkumyan et al., 2022). Interestingly, while studies by Warden et al. (2020) have implicated microglia in chronic alcohol induced increase in glutamatergic transmission in the CeA_*M*_ of alcohol-dependent mice, the study by Melkumyan et al. (2022) did not find the involvement of microglia in the mechanism of action of acute alcohol in alcohol naïve mice. Together, these findings may suggest that astrocytes are predominant drivers of acute effect of alcohol in naïve animals, while microglia may become more involved following chronic alcohol exposure; however, more studies are needed to investigate this hypothesis (see “Modulators of Proposed Circuit – Neuroinflammation” Section below for more details). It is worth noting that most studies conducted in the CeA_*L*_ were conducted on mice, while studies in the CeA_*M*_ were mostly conducted on rats. To our knowledge there has only been one study comparing evoked glutamatergic activity of CeA_*M*_ and CeA_*L*_ subdivisions in male and female Wistar rats, showing 44 mM alcohol decreases BLA-evoked glutamatergic transmission in both the CeA_*L*_ and CeA_*M*_ subdivisions, with no baseline differences in stimulus-response activity (Logrip et al., 2017).

### Sex Differences

It is known that estrogen and estrogen receptors are present in the amygdala and play a role in anxiety and depressive behaviors (Walf and Frye, 2006). Additionally, estrogen receptors have been shown to co-localize with NMDA receptors in the amygdala (Kia et al., 2002), making glutamatergic transmission in the CeA sensitive to changes in the estrous cycle. Examination of sex differences on alcohol effects in CeA glutamatergic transmission has been limited. Kirson et al. (2018) compared male and female msP and Wistar rats and found some strain specific sex differences, with male, but not female, msP rats showing a reduction in the probability of glutamate release after alcohol application. There were no significant differences in the response to alcohol between the estrous cycle of the rats, however, diestrus msP rats were unaffected by alcohol, while estrus phase Wistar rats had a larger, although not significant, response to alcohol compared to diestrus Wistar rats. Studies in Wistar rats comparing the response of CeA_*L*_ and CeA_*M*_ cells to acute 44 mM alcohol application in males and females saw no significant differences in stimulus-response properties (Logrip et al., 2017). However, in the CeA_*M*_, and not in the CeA_*L*_, alcohol increased evoked response in proestrus cycle in females, but decreased the evoked response in the estrus cycle (Logrip et al., 2017). Lastly, a recent study by Bloodgood et al. (2021) found that alcohol drinking through three cycles of drinking in the dark procedure alters excitability of PDYN neurons in the CeA in a sex dependent manner with no differences in spontaneous synaptic transmission. Specifically, the study showed that after alcohol drinking, male Pdyn*^IRES–Cre^*::Gt(ROSA26)Sor*^loxSTOPlox–L10–GFP^* mice fired more action potentials compared to male non-alcohol drinking mice, while female alcohol drinking mice had a non-significant reduction in action potential firing compared to female non-alcohol drinking mice (Bloodgood et al., 2021). Overall, it is important to further study sex differences to understand the mechanism of alcohol on the glutamatergic transmission in the CeA subregions.

## Modulators of Proposed Circuit

The varying effect of alcohol on the glutamatergic and GABAergic transmission in the subregions of the CeA may be mediated through neuroinflammation and heterogenous neuronal populations containing various neuropeptides and neuromodulators (Gilpin and Roberto, 2012). Below is an overview of some the most well-studied CeA circuit modulators in terms of alcohol research, but future studies at both the preclinical and clinical levels will be needed to establish which of the myriad of possible CeA neuromodulators may be targeted as pharmacotherapeutics for AUD.

### Neuroinflammation

Neuroinflammation has been heavily implicated in AUD, and studies have shown that neuroinflammatory cells and signals play an important role in the effect of alcohol on glutamatergic and GABAergic transmission in the CeA (Table 3, Bajo et al., 2019; Patel et al., 2019; Warden et al., 2020; Melkumyan et al., 2022). Studies from our lab have shown that inhibition of astrocytes, but not microglia, attenuates acute alcohol-induced increases in glutamatergic transmission in the CeA_*L*_, suggesting a critical role for astrocytes in the mechanism of alcohol actions in the CeA (Melkumyan et al., 2022). Studies have shown the involvement of microglia in the effect of chronic alcohol, as microglial depletion resulted in reduction of both glutamatergic and GABAergic transmission in CeA of alcohol dependent mice (Warden et al., 2020). However, microglia depletion does not regulate acute alcohol-induced sedation or motor incoordination, or escalation and maintenance of chronic voluntary alcohol intake (Warden et al., 2021). Additional studies show no changes to microglia density or morphology in the CeA after binge-like alcohol drinking in the dark paradigm (Nelson et al., 2021). Transcriptomic analysis showed that systemic downregulation of microglia does not result in changes in alcohol intake, however, the combination of the downregulation of microglia and chronic alcohol resulted in an upregulation of reactive astrocyte genes (Warden et al., 2021). A recent gene expression study has also shown that astrocyte-specific genes were differentially expressed in the CeA after chronic intermittent alcohol exposure compared to control group (Kisby et al., 2021). Overall, the aforementioned studies suggest that alcohol exposure can increase the expression of astrocyte-specific genes and that CeA astrocytes may be critical regulators of initial alcohol effects on CeA circuit function and behavior. Microglia activity may be critical to effects related to chronic alcohol exposure and may interact with astrocytic mechanisms. More studies are needed to explore the role of astrocytes and microglia in acute and chronic alcohol. It is worth noting that a recent clinical study showed that minocycline, a microglia inhibitor, did not reduce alcohol craving or seeking in heavy drinkers (Petrakis et al., 2019), however, more targeted therapies may be needed.

**TABLE 3:** The alcohol-related functions of the neuroinflammatory system components.

Neuroinflammatory system components	Function (alcohol related)
Astrocytes	Mediates the mechanism of acute and chronic alcohol on glutamatergic transmission in the CeA (Kisby et al., 2021; Melkumyan et al., 2022)
Microglia	Mediates the mechanism of chronic alcohol on glutamatergic and GABAergic transmission in the CeA with no effect on alcohol drinking (Warden et al., 2020, 2021; Nelson et al., 2021)
Pro-inflammatory cytokines	Pro-inflammatory cytokines generally increase CeA GABAergic transmission;
	CeA pro-inflammatory cytokines are generally increased after alcohol (Bajo et al., 2015, 2019; [Bibr B111][Bibr B125])
Anti-inflammatory cytokines	Reduced levels of anti-inflammatory cytokines after alcohol exposure (Qin et al., 2008)

In terms of which neuroimmune signals may mediate astrocyte and microglia mechanism of alcohol on neuronal signaling, studies have found increased systemic and brain pro-inflammatory cytokine levels after chronic alcohol exposure, including TNFα and IL-1β (Qin et al., 2008; Patel et al., 2019). In addition to increasing the cytokine levels on its own, alcohol also potentiates the effect of lipopolysaccharide (LPS) – a bacterial endotoxin commonly used to induce neuroinflammatory response – to increase levels of TNFα, IL-1β, and MCP-1 (Qin et al., 2008). Lastly, alcohol reduced levels of the anti-inflammatory cytokine IL-10 in the brain (Qin et al., 2008), suggesting an overall increase in neuroinflammatory response after alcohol treatment.

In the CeA specifically, neuroimmune signaling through IL-1β has been shown to alter GABAergic transmission in the CeA_*M*_ at pre- and post-synaptic sites through a potentially different mechanism than alcohol (Bajo et al., 2015; Patel et al., 2019). Even though IL-1β does not alter alcohol-induced facilitation of GABA release, chronic alcohol exposure increases IL-1β levels in neurons and microglia (Patel et al., 2019). A recent study looked at the effect of myeloid differentiation primary response protein (MyD88), which has been implicated in alcohol-related behaviors, on IL-1β and alcohol modulation of GABAergic transmission in the CeA (Bajo et al., 2019). It was found that in MyD88 knockout mice, there was a more robust increase in sIPSC frequency after 100 mM alcohol exposure, with no effect on 44 mM alcohol or IL-1β activity. Studies have shown increased GABAergic transmission in the CeA in mice with elevated IL-6 levels (Roberts et al., 2019), similar to the increase in GABAergic transmission after alcohol exposure (see Section “GABAergic Transmission” above). Additionally, our recent studies (Melkumyan et al., 2022) show that neuroimmune stimulation with LPS increases CeA_*L*_ glutamatergic transmission, particularly when microglia are inhibited, further suggesting a critical role for astrocytes in modulation of CeA function. Overall, further studies are needed to elucidate the role cytokine activation on the mechanism of alcohol action on CeA neurotransmission.

### Cannabinoid Signaling

The endocannabinoid system is involved in alcohol drinking and mitigates alcohol induced increase in neuroinflammatory signaling (Table 4, Zhou et al., 2016; García-Baos et al., 2021). The endocannabinoid system is comprised of endocannabinoids, enzymes responsible for synthesis and metabolism of endocannabinoids, and the cannabinoid receptors type 1 (CB1R) and type 2 (CB2R), both of which are G-protein coupled receptors expressed widely in the central and peripheral tissues (Ramikie and Patel, 2012; Lu and Mackie, 2016). Global knockout of the endocannabinoid metabolism enzyme fatty acid amide hydrolase (FAAH) in male, but not female, mice results in increased alcohol consumption and preference and decreased depressant effect of alcohol, with no changes in saccharin preference or intake (Blednov et al., 2007). Conversely, a study in Wistar rats saw no effect of systemic injection of a FAAH inhibitor on operant self-administration of alcohol (Cippitelli et al., 2008). Interestingly, inhibition of CB1R activity with rimonabant reduced operant alcohol responding in both Wistar and msP rats (Cippitelli et al., 2005, 2008). Similarly, male and female CB1R knockout mice consumed less alcohol and had reduced preference for alcohol compared to control mice with no changes in sucrose and quinine consumption (Naassila et al., 2004). These results suggested that genetic and pharmacologic alteration of endocannabinoid signaling leads to a reduction in alcohol consumption and preference.

**TABLE 4:** The alcohol-related functions of the endocannabinoid system components in the CeA.

Endocannabinoid system components	Function (alcohol related)
Fatty acid amide hydrolase (FAAH)	Inverse relationship between FAAH level and alcohol consumption and preference in sex and strain specific manner (Blednov et al., 2007; Cippitelli et al., 2008)
Cannabinoid receptor 1 (CB1R)	Activation decreases glutamate release in CeA in a sex and strain specific manner (Ramikie et al., 2014; Kirson et al., 2018)
	Activation reverses effect of acute alcohol-induced increase in GABAergic transmission in CeA_M_ (Roberto et al., 2010)
Cannabinoid receptor 2 (CB2R)	Inverse relationship with alcohol preference and vulnerability/withdrawal (Ortega-Álvaro et al., 2015; Serrano et al., 2018)
2-arachidonoylglycerol (2-AG) and anandamide (AEA)	Lower baseline level of 2-AG, but not of AEA, in alcohol exposed rats, (Serrano et al., 2018)
	Decreasing 2-AG and AEA levels during alcohol abstinence with recovery of 2-AG levels after alcohol reinstatement (Serrano et al., 2018)
Cannabidiol and delta-9-tetrahydrocannabinol (CBD:THC)	Reduced alcohol consumption, motivation, alcohol-induced hypothermia and neurotoxicity with CBD, not THC, use (Viudez-Martínez et al., 2018; Karoly et al., 2021)

CB1Rs are more widely expressed in the central nervous system and neurons, while CB2Rs are predominantly expressed in immune and hematopoietic systems (Kunos, 2020). Downregulation in endocannabinoid signaling and endocannabinoid concentrations have been shown in the CeA of alcohol dependent rats (Sanchez-Marin et al., 2017; Serrano et al., 2018). Serrano et al. (2018) found reduced baseline 2-arachidonoylglycerol (2-AG), but not anandamide (AEA), levels in alcohol exposed Wistar rats, with abstinence inducing a further reduction in both 2-AG and AEA levels. It has been suggested that after alcohol withdrawal, the decrease in the extracellular concentration of 2-AG, but not AEA, is associated with enhanced glutamate release, an effect that is recovered after alcohol is reinstated (Serrano et al., 2018; Suárez et al., 2020). CB1R activation has been shown to decrease glutamate release in the CeA (Ramikie et al., 2014) and CB1R agonist WIN55212-2 has been shown to act in a sex- and strain-specific manner to decrease alcohol induced changes in glutamatergic transmission in the CeA (Kirson et al., 2018). Additionally, the endocannabinoid system has been shown to regulate GABAergic transmission in the CeA after alcohol application (Roberto et al., 2010). A study by Roberto et al. (2010) showed that application of the CB1R agonist WIN55212-2 reverses the effect of acute alcohol-induced increases in GABAergic transmission in the CeA_*M*_. Interestingly, it was found that co-application of the CB1R antagonist and alcohol leads to a further increase in GABA transmission compared to the antagonist alone (Roberto et al., 2010). This data suggests that the alcohol effects on CeA GABAergic transmission are limited by CB1R mechanism. The effect of CB1R on glutamatergic and GABAergic transmission may also be mediated by changes in neuroinflammatory signaling as astrocytic CB1Rs reduce neuroimmune activity and participate in gliotransmission.

Since CB2Rs are less expressed in neuronal populations, they have not been as widely studied as CB1Rs in terms of alcohol effects. Studies have shown that CB2Rs may be involved in alcohol-seeking behaviors, with CB2R knockout mice increasing preference and vulnerability to alcohol consumption (Ortega-Álvaro et al., 2015). Additionally, in the amygdala, there was a reduction of the CB2R gene expression 24 h into alcohol withdrawal (Serrano et al., 2018). The mechanism of action of CB2Rs on alcohol-related behaviors may occur through astrocytic and microglial activation since these receptors are commonly found on neuroimmune cells (Crowe et al., 2014). These findings may have direct clinical relevance as a study in Japanese individuals showed an association between polymorphisms in the CB2R coding gene and AUD (Ishiguro et al., 2007).

Given that acute and chronic alcohol may interact with the endocannabinoid system, interest in the use of phytocannabiniods (cannabinoids from plant products) and exogenous cannabinoids (pharmaceutically produced cannabinoids) for treatment of AUD has increased. Phytocannabinoids and exogenous cannabinoids have been approved in some areas for medical treatment of mental health disorders commonly associated with AUD, such as anxiety disorders and post-traumatic stress disorder. Two highly studied cannabinoids are cannabidiol (CBD) – a non-euphorigenic phytocannabinoid – and delta9-tetrahydrocannabinol (THC) – the main euphorigenic phytocannabinoid. CBD can act as a non-competitive negative allosteric modulator of CB1R and CB2R, especially in the presence of THC, leading to a reduction of the psychoactive effects of THC (Laprairie et al., 2015; Karoly et al., 2021). Observational studies in humans have shown that CBD and alcohol co-users tend to drink less alcohol than THC users or THC and CBD users, suggesting that CBD may reduce alcohol drinking in humans (Karoly et al., 2021). Additionally, studies showed that CBD can reduce alcohol consumption, alcohol motivation, and alcohol-induced hypothermia in C57Bl/6J mice (Viudez-Martínez et al., 2018). The mechanism of CBD on alcohol intake is proposed to be through effects such as blocking microglial activation, inhibiting expression of proinflammatory miRNA associated with toll-like receptors (TLRs) and NF-κB signaling, and reducing the expression of pro-inflammatory cytokines like IL-1β and TNFα through activation of peroxisome proliferator-activated receptor-γ (PPAR-γ) (Esposito et al., 2011; Martín-Moreno et al., 2011; Patricio et al., 2020; Ożarowski et al., 2021), targets previously shown to be modulated by chronic alcohol exposure. Studies have shown that CBD administration leads to a reduction of alcohol-induced neurotoxicity in rats (Hamelink et al., 2005). Overall, the interaction between endocannabinoid system with alcohol-related behaviors, neurotransmission in the CeA, and neuroinflammation makes the endocannabinoid system a potential target for AUD treatments.

### Neuromodulators/Neuropeptides

Neuropeptides play an important modulatory role throughout the brain and the nervous system in general. In the CeA specifically, neuropeptides are involved in all of the actions of the CeA, including stress and anxiety (Gilpin, 2012b; Gilpin et al., 2015; Pomrenze et al., 2019), pain (Neugebauer et al., 2020), addiction (Gilpin, 2012b; Gilpin et al., 2015), and overall activity of the CeA (Silberman and Winder, 2015). To better understand the mechanisms of action of alcohol on the CeA and its specific subregions, it is important to look at the neuropeptide composition of these subregions and their role in alcohol use (Table 5). Below, we will briefly touch on some of the common peptides and their roles in AUD (for more comprehensive reviews please see Gilpin, 2012b; Silberman and Winder, 2013; Gilpin et al., 2015; Walker, 2021).

**TABLE 5:** Commonly studied neuropeptides in the CeA modulating alcohol related effects.

Peptide	Primary subregion	Projections	Connections with other peptides	Function (alcohol related)
Corticotropin releasing factor (CRF)	CeA_L_	Inputs from PVN, local microcircuitry with CRF+ cells, projections to nucleus accumbens	Co-localized with GABA	CRFR1 and CRFR2 required to block effect of alcohol on glutamatergic transmission in CeA (Silberman et al., 2015) CRFR1 antagonists reduce alcohol consumption (Finn et al., 2007; Lowery-Gionta et al., 2012; Weera et al., 2020; Walker, 2021)
Neuropeptide Y (NPY)	CeA_M_	Projections to and from intercalated neurons, BNST	Co-localization with SST	Lower baseline levels of NPY in alcohol-preferring rats/mice (Ehlers et al., 1998; Hayes et al., 2005; Robinson and Thiele, 2017) NPY infusion into CeA reduces alcohol intake (Zhang et al., 2010)
Neurotensin (Nts)	CeA_M_ (∼60%), CeA_L_ (∼35%)	Reinforcing projections to parabrachial nucleus and BNST	Co-express CRF or SST	Alcohol consumption activates Nts neurons in CeA_L_ (Torruella-Suárez et al., 2020) PBN to CeA projecting Nts neurons promote alcohol drinking (Torruella-Suárez et al., 2020)
				Ablation of Nts in CeA decreases alcohol consumption (Torruella-Suárez et al., 2020).
Nociceptin/Orphanin FQ	CeA_L_ and CeA_M_	Projections to BNST, hippocampus, nucleus accumbens	Overlap with SST, serotonin, and PKCd	Reduces glutamatergic activity in the CeA of naïve and alcohol dependent rats (Kallupi et al., 2014) Reduced GABAergic transmission in the CeA and blocked alcohol induced increase in GABAergic transmission (Roberto and Siggins, 2006)
Orexin/Hypocretin	Mostly in CeA_L_, also in CeA_M_	Projections from the hypothalamus	Co-localized with glutamate	Intra-CeA injections of orexin 1 receptor antagonist reduce alcohol consumption (Olney et al., 2017) Orexin depolarizes CeA_M_ neurons (Bisetti et al., 2006)
Protein Kinase Cd (PKCd)	CeA_L_, CeA_C_, projections to CeA_M_	Inhibit output neurons in CeA_M_	Inhibit SST+ cells	May act on alcohol effects on GABAergic transmission through CRF mediated mechanisms (Gilpin et al., 2015)
Somatostatin (SST)	CeA_L_	Inhibit CeA_L_ non-SST neurons	Inhibit PKCd cells	Excitation and silencing in prelimbic cortex leads to reduction in alcohol drinking in males and females (Dao et al., 2021)
			Overlap with tachykinin 2 cells	
Substance P	Substance P in CeA_C_	Induces GABA release in CeA_M_	Neurokinin-1 receptor (NK-1R)	Substance P reduces alcohol responding (Yang et al., 2009)
	NK-1R in CeA_L_			NK-1R overexpression increases alcohol self-administration (Nelson et al., 2019)
				Substance P/NK-1R system involved in alcohol dependence and withdrawal in an inverted-U shaped dose-dependent manner (Khom et al., 2020)

#### Corticotropin Releasing Factor

One of the most studied neuropeptides in the CeA in terms of AUD is corticotropin releasing factor (CRF). CRF is a neuropeptide encoded from the *crh* gene that is highly involved in stress and fear response and is heavily implicated in alcohol use disorder (Koob, 2010; Silberman and Winder, 2015; Silberman et al., 2015; Varodayan et al., 2017a; Agoglia and Herman, 2018; de Guglielmo et al., 2019). CRF has two G-protein coupled receptors, CRFR1 and CRFR2. In the CeA_*M*_ tonic CRFR1 activity enhances glutamate release, while tonic CRFR2 activity inhibits glutamate release (Varodayan et al., 2017a). Additionally, application of CRF in the CeA increases GABA release in CRFR1, but not CRFR2, dependent manner (Nie et al., 2004; Agoglia and Herman, 2018). Further, acute alcohol-induced increase of GABA release in CeA_*M*_ was abolished after CRFR1 knockout (Nie et al., 2004), suggesting that CRFR1 receptors are responsible for the alcohol effect on GABAergic transmission in the CeA_*M*_. Both CRFR1 and CRFR2 antagonists are necessary to block the effect of alcohol to increase glutamatergic transmission in the CeA_*L*_ (Silberman et al., 2015). In msP rats exposed to two-bottle choice paradigm, CRF application led to an increase in spontaneous action potential-independent GABAergic activity, similar to naïve msP rats, while CRFR1 antagonist abolished this increase in two-bottle choice exposed msP rats (Herman et al., 2013). Behaviorally, CRFR1 receptor antagonists reduce both alcohol consumption and alcohol withdrawal induced irritability-like behaviors in animal models (Finn et al., 2007; Lowery-Gionta et al., 2012; Kimbrough et al., 2017; Weera et al., 2020; Walker, 2021). Stimulation of the neurons projecting from CeA to nucleus accumbens leads to reduction in binge-like alcohol drinking, and this effect is mediated by CRFR1 activity (Borrego et al., 2022). Additionally, it is known that CeA CRF+ neurons show increased activity compared to non-CRF cells during binge drinking sessions in the drinking in the dark paradigm (for more details, see “Circuit Manipulation and Alcohol Consumption” section below, Aroni et al., 2021). However, the reduction in alcohol consumption through antagonism of CRF+ and CRFR1 mechanisms has not yet translated to human treatment of AUD, as initial clinical studies using CRFR1 antagonists did not lead to a reduction in alcohol craving (Kwako et al., 2015; Schwandt et al., 2016). Therefore, more preclinical and clinical studies may be needed to understand the disparities between the animal models and human studies.

#### Neuropeptide Y

Neuropeptide Y (NPY) is highly expressed in various brain regions, including the amygdala (Dumont et al., 1990; Gustafson et al., 1997), and is involved in alcohol drinking (Ehlers et al., 1998; Robinson and Thiele, 2017). Dense populations of NPY-GFP-positive neurons were observed in the CeA_*M*_ with a few neurons in the CeA_*L*_ (Wood et al., 2016). NPY has at least five receptor subtypes, of which the best characterized are Y1, Y2, and Y5 receptors. NPY acts postsynaptically through Y1 receptors (Y1Rs) and presynaptically through Y2Rs. Y2Rs act as autoreceptors, inhibiting the release of NPY, but can also act as heteroreceptors inhibiting the release of glutamate and GABA (Sparrow et al., 2012), making these receptors a good target for studying alcohol actions on transmission in the CeA. In alcohol preferring P rats, there was a decrease in NPY-like immunoreactivity in the hippocampus, frontal cortex, and amygdala compared to non-preferring rats (Ehlers et al., 1998). NPY infusion directly into the CeA reduces alcohol intake in the alcohol-preferring P rat line (Zhang et al., 2010). Additionally, alcohol-preferring C57Bl/6J mice have lower baseline levels of NPY compared to alcohol-non-preferring DBA/2J mice (Hayes et al., 2005; Robinson and Thiele, 2017). Overall, NPY expression level is negatively correlated with alcohol preference.

At the circuit level, NPY has been shown to prevent and reverse alcohol-induced increases in GABAergic transmission through a presynaptic mechanism mostly mediated by the Y2 receptor (Gilpin et al., 2011). Three cycles of binge-like alcohol drinking led to an increase in NPY-induced GABAergic activity in the CeA, suggesting that binge-like alcohol drinking increases NPY modulation of GABAergic activity (Sparrow et al., 2012). Interestingly, both systemic and intra-CeA inhibition of Y2 receptors did not influence alcohol self-administration in alcohol dependent and non-dependent rats (Kallupi et al., 2014c). Y1 receptors have also been implicated in alcohol consumption, with the inhibition of lateral habenula projecting Y1 receptor containing CeA neurons leading to a reduction in binge-like alcohol consumption, with no effect on sucrose (Companion et al., 2022). Even though the inhibition of Y1 receptor containing CeA neurons projecting to the lateral habenula had a behavioral effect, there were no changes to the inhibitory currents in these neurons after one 4-day cycle of binge-like alcohol consumption (Companion et al., 2022). Overall, these findings show the involvement of NPY on alcohol related behavioral and physiological effects (for a more comprehensive review of the effect of NPY and alcohol please see Gilpin, 2012a,b; Robinson and Thiele, 2017).

#### Neurotensin

The largest population of neurotensin (Nts) neurons in the CeA is found in the CeA_*M*_ (∼60%), although the CeA_*L*_ has a large population of Nts neurons as well (∼35%) (McCullough et al., 2018). Nts commonly binds to its GPCR receptors NTSR1 and NTSR2. NTSR1 receptors are commonly found in neurons, while NTSR2 receptors are more present in glia (Yamauchi et al., 2007; Woodworth et al., 2018; Torruella-Suárez and McElligott, 2020). Nts neurons commonly co-express with CRF or somatostatin, particularly in the CeA_*L*_, making Nts a strong candidate in mediating some of alcohol effects on CeA transmission. Supporting this hypothesis, a recent study showed that (1) alcohol consumption activates the Nts neurons in the CeA_*L*_, (2) optogenetic stimulation of the CeA Nts+ projections to parabrachial nucleus (PBN) promotes consumption of alcohol drinking, and (3) the ablation of Nts neurons in the CeA decreases alcohol consumption in two-bottle choice test (Torruella-Suárez et al., 2020). In addition to their projections to the PBN, Nts neurons also project to the BNST – a region heavily implicated in alcohol and substance use (Snyder and Silberman, 2021) – and modulate inhibitory transmission to the CeA from the BNST through NTSR1 and kappa opioid receptors (Normandeau et al., 2018; Torruella-Suárez and McElligott, 2020). Activation of these receptors can in turn differentially modulate GABAergic transmission in the CeA (Normandeau et al., 2018). Even though the circuitry of Nts neurons projecting from the CeA to the BNST has not been widely studied in terms of alcohol use, Nts neurons in the CeA are a subpopulation of CRF neurons, which have been heavily implicated in alcohol use [see above Section “Corticotropin Releasing Factor (CRF)”]. Nts neurons in the CeA seem to play a significant role in alcohol effects, however, more extensive research is required to determine if this system may be an effective therapeutic target for AUD.

#### Nociceptin

Nociceptin acts through the nociceptin/orphanin FQ Opioid Peptide Receptor (NOP) and can have effects on alcohol-related behaviors (Witkin et al., 2014; Walker, 2021). Oral administration of NOP antagonist led to a significant reduction in alcohol intake through the two-bottle choice paradigm, particularly in females (Borruto et al., 2020). Additionally, genetic deletion of NOP led to a significant reduction in alcohol self-administration (Kallupi et al., 2017). On the other hand, agonists of NOP reduced self-administration of alcohol and alcohol drinking in alcohol-preferring P rats, in addition to reductions in food and water consumption, suggesting a non-specific effect of NOP on alcohol motivation (Witkin et al., 2014; de Guglielmo et al., 2015). These findings suggest that NOP is involved in alcohol consumption and motivation, although it may act on overall appetitive behaviors as opposed to alcohol consumption alone.

Intracerebroventricular (ICV) injections of nociceptin in msP rats led to a significant increase in alcohol intake acutely but led to a progressive decrease in alcohol intake when administered subchronically within a 9 days period (Ciccocioppo et al., 1999). Similarly, 6 days of consecutive ICV injections of nociceptin led to a reduction in alcohol self-administration in msP rats, with no effect on Wistar rats (Economidou et al., 2008). In the CeA specifically, the effect of nociceptin on alcohol drinking was further confirmed by an upregulated expression of nociceptin and NOP mRNA in msP rats compared to Wistar rats (Economidou et al., 2008). Confirming the relationship between NOP and alcohol preference, microinjections of NOP antagonist into the CeA led to a reduction in two-bottle choice alcohol intake, with no changes in water or food intake, suggesting that NOP in the CeA has inhibitory effects on alcohol consumption (Borruto et al., 2020). These findings suggest that NOP systemically may be involved in appetitive behaviors, however, in the CeA, NOP has specific effects on alcohol consumption [see Witkin et al. (2014) and Walker (2021) for further review of nociceptin system].

In terms of neurotransmission in the CeA, nociceptin reduced evoked and spontaneous glutamatergic activity in the CeA of naïve and chronic alcohol exposed Wistar rats and reduced the probability of glutamate release (Kallupi et al., 2014b). Nociceptin application also blocked alcohol induced decrease in glutamatergic transmission in the CeA_*M*_ (Kallupi et al., 2014b). Chronic alcohol exposure did not alter nociceptin signaling in the CeA_*M*_, as nociceptin significantly decreased evoked glutamatergic activity with no further alteration after chronic alcohol exposure (Kallupi et al., 2014b). These findings suggest that nociceptin and alcohol both affect glutamatergic transmission in the CeA_*M*_, likely at the presynaptic site. Nociceptin reduces GABAergic transmission in the CeA presynaptically, and blocks the alcohol induced increase in GABAergic transmission in naïve and alcohol-dependent rats, potentially through interactions with CRF-mediated mechanisms (Roberto and Siggins, 2006; Cruz et al., 2012; Ciccocioppo et al., 2014). Similarly, activation of NOP through a selective agonist leads to reductions in mostly presynaptic GABAergic transmission in the CeA, and inhibits the alcohol induced increase in GABAergic transmission (Kallupi et al., 2014a). Although further studies need to be conducted to understand the exact mechanism of action of nociceptin on CeA glutamatergic and GABAergic activity, the nociceptin system may be a novel therapeutic target for AUD to be addressed in future research.

#### Orexin/Hypocretin

Neurons that produce orexin/hypocretin are located in the hypothalamus and send projections to the orexin 1 and orexin 2 receptors in various brain regions, including the CeA (Olney et al., 2017). Orexin A (hypocretin 1) has been shown to occur in the neuronal cell bodies of the hypothalamus, while orexin B (hypocretin 2) has been identified in the CeA and BNST, particularly in the CeA_*L*_ (Ciriello et al., 2003). Studies have shown the involvement of orexin in alcohol consumption [for an in-depth review of the effect of orexin on alcohol-seeking see (Lawrence, 2010 and Matzeu and Martin-Fardon, 2022)]. Clinical studies have shown an inverse relationship between orexin A receptor and the severity of withdrawal symptoms in patients with AUD (Bayerlein et al., 2011; Matzeu and Martin-Fardon, 2022). In preclinical models, injections of orexin receptor antagonist reduced cue-induced reinstatement of alcohol drinking and operant responding to alcohol in alcohol preferring P rats (Lawrence et al., 2006). In the CeA specifically, intra-CeA treatment with orexin 1 receptor antagonist reduced binge-like alcohol consumption, with no effect on sucrose consumption (Olney et al., 2017). Treatment with orexin 2 receptor selective antagonist seemed to block the increase in alcohol drinking instead of reducing the drinking behavior (Olney et al., 2017). In terms of potential mechanisms, CeA_*M*_ neurons are strongly depolarized by orexin through the activation of orexin 2 receptors, indicating the direct effect of these neurons on the CeA (Bisetti et al., 2006). Further studies are needed to explore the effect of CeA orexin containing neurons on CeA neurocircuit activity and alcohol-directed behaviors.

#### Protein Kinase Cδ and Somatostatin Microcircuit

The CeA_*L*_ consists of GABAergic medium spiny neurons that express protein kinase Cδ (PKCδ) and somatostatin (SST) (Babaev et al., 2018). These cells form local inhibitory microcircuits and have been shown to play a role in fear and anxiety behaviors (Babaev et al., 2018; Walker, 2021). Specifically, SST+ and PKCδ+ neurons are mutually inhibiting, and activation of SST neurons leads to inhibition of PKCδ neurons projecting to the CeA_*M*_, resulting in a disinhibition of the CeA_*M*_ outputs to promote anxiety-like behaviors (Haubensak et al., 2010; Li et al., 2013; Penzo et al., 2014).

The SST neurons are the largest population of CeA_*L*_ cells overlapping with tachykinin 2 (TAC2) cells – cells present in the CeA_*L*_ and CeA_*M*_ that are necessary for fear learning (McCullough et al., 2018). SST neurons inhibit non-SST neurons in the CeA_*L*_, but they do not send projections to the CeA_*M*_ or inhibit CeA_*M*_ neurons. Contrary to the projections of SST neurons, the PKCδ neurons in the CeA_*L*_ have been shown to inhibit the output neurons in the CeA_*M*_ (Haubensak et al., 2010; McCullough et al., 2018; Walker, 2021).

Since CeA_*L*_ PKCδ neurons regulate CeA_*M*_ outputs and the expression of anxiety-like behaviors, and since acute and chronic alcohol has been shown to affect anxiety-like behaviors (Silberman and Winder, 2015; Walker, 2021), it is possible that PKCδ may play a role in alcohol induced changes in GABAergic and glutamatergic transmission in the CeA that drive continued alcohol intake in AUD, although this has not been directly tested. In addition to PKCδ, PKCε and PKA have been shown to play a role in alcohol modulation of GABAergic transmission in the CeA through CRF mediated mechanisms (Gilpin et al., 2015). Other work indicates SST neurons play an important role in alcohol intake as both chemogenetic activation and silencing of SST neurons in the prelimbic cortex has shown to reduce alcohol binge drinking in male and female mice (Dao et al., 2021). However, the exact mechanisms by which CeA PKCδ and SST microcircuits alter glutamatergic and GABAergic transmission in the CeA and modulate alcohol-directed behaviors is not fully understood and needs to be further explored as potential novel treatment options for AUD.

#### Substance P and Neurokinin

Substance P and its molecular target, neurokinin-1 receptor (NK-1Rs) are present in the amygdala, with NK-1Rs being most widely present in the CeA_*L*_ and substance P being most widely present in CeA_*C*_ (Cassell and Gray, 1989; Khom et al., 2020). Substance P mRNA levels are lower in alcohol-preferring P rats compared to non-preferring rats, while intra-CeA infusion of substance P decreases operant alcohol responding in a dose-dependent manner in alcohol-preferring P rats (Yang et al., 2009). Interestingly, a study in 2019 uncovered the role of NK-1R in alcohol preferring rats, with NK-1R antagonist inhibiting yohimbine induced alcohol reinstatement (Nelson et al., 2019), with an overexpression of NK-1R leading to an increase in alcohol self-administration (Nelson et al., 2019). A recent study by Khom et al. (2020) showed that substance P induces GABA release in the CeA_*M*_ through interaction of G protein-coupled inwardly rectifying K^+^ channels. Interestingly, the study found that chronic intermittent alcohol exposure significantly reduces substance P and NK-1R levels as observed through immunohistochemical staining in the CeA_*M*_ (Khom et al., 2020), which raises questions on the expression of NK-1R after alcohol exposure, since previous studies showed an overexpression of the NK-1R in increased alcohol self-administration (Nelson et al., 2019). Substance P application did not alter GABA transmission in the CeA_*M*_ in dependent rats compared to naïve rats at low (1 and 10 nM) and high (300 nM) concentrations, but had a significantly sensitized effect to intermediate concentrations of substance P (30 and 100 nM), suggesting an inverted U concentration effect curve. Additionally, in naïve and dependent rats, the increase of sIPSC frequency after acute 44 mM alcohol application was sensitized after 100 nM substance P application, and this effect remained after alcohol withdrawal (Khom et al., 2020). These findings suggested that substance P/NK-1R system is involved in alcohol dependence and withdrawal in potentially an inverted-U shaped dose-dependent manner. The results from the aforementioned studies need to be explored further to understand the role of the substance P/NK-1R system on alcohol effects in the CeA.

## Circuit Manipulation and Alcohol Consumption

The electrophysiological studies point to the involvement of the CeA in alcohol effects, however, it is important to consider *in vivo* manipulations of the CeA neurons when assessing the interaction between CeA neuronal activation and behavioral outcomes, such as alcohol consumption. It is worth noting that viral or pharmacologic manipulations of the CeA *in vivo* are limited in their approach, as currently, intracranial injections are not typically able to target the specific subregions of the CeA. More selective viral and pharmacologic approaches will be needed in future studies. Below is a brief summary of some of the literature on the relationship between alcohol consumption and *in vivo* CeA neuronal activity.

A recent study by Haaranen et al. (2020) has shown that manipulation of the CeA neurons of Alko Alcohol Accepting rats through inhibitory and excitatory DREADDs has a differential effect on alcohol intake through the two-bottle choice paradigm. Activation of CeA neurons through excitatory DREADDs led to a reduction in alcohol drinking compared to baseline, while inhibition of CeA neurons had no effect on alcohol drinking (Haaranen et al., 2020). Another study found that inactivation of CeA neuronal ensembles, particularly CRF neurons, reduces alcohol drinking in the two-bottle choice paradigm in non-dependent rats and reduces alcohol self-administration in dependent rats (de Guglielmo et al., 2016, 2019). These studies suggest that the CeA is highly involved in the modulation of alcohol drinking. Since the neuronal populations of the CeA subregions are highly heterogenous, it is important to parse out the effects of different cell types on alcohol drinking.

Studies have shown that intra-CeA injections of a kappa opioid receptor antagonist reduces binge-like alcohol drinking during a 4-h drinking in the dark session (Anderson et al., 2019). Data from the same group showed that DREADD inactivation of dynorphin containing neurons in the CeA led to a reduction in binge-like alcohol consumption, an effect that was reversed by a kappa opioid receptor agonist (Anderson et al., 2019), suggesting that dynorphin and kappa opioid receptors in the CeA are necessary for the regulation of alcohol consumption. A recent study by Aroni et al. (2021) used *in vivo* electrophysiological methods to study the role of CRF neurons on binge-like alcohol consumption in CRF-Cre mice. The group showed that pre-lick activated CRF cells had a higher firing activity than other CRF cells, with a steadily increasing firing rate over the length of the drinking experiment and in later drinking sessions compared to earlier ones (Aroni et al., 2021). Additionally, CeA CRF neurons did not have an increased firing rate compared to non-CRF cells in response to sucrose, indicating a preferential increase in activity in response to alcohol (Aroni et al., 2021). This data suggested that a subtype of CRF cells in the CeA may be modulating binge-like alcohol consumption over time. The involvement of CeA CRF system in regulating drinking behaviors is supported by Lowery-Gionta et al. (2012) who showed that intra-CeA injection of a selective CRF1R antagonist in male C57Bl/6J mice led to a reduction in binge-like alcohol consumption. These studies provide insight into the involvement of the heterogenous cellular populations in the CeA in alcohol consumption. Further studies are needed to parse out the differences in these neuronal populations and understand how these neurons alter alcohol consumption and how alcohol consumption alters the glutamatergic and GABAergic activity of these neurons.

## Summary

The differential effect of alcohol on CeA neurotransmission is likely due to the actions of the subregion specific neuromodulators, neuropeptides, and neuroimmune cells. It is likely that the CRF1R and CRF2R modulate glutamatergic transmission in the CeA_*L*_, increasing activity of CeA_*L*_ neurons resulting in enhanced GABAergic signaling in the CeA_*M*_. CRF expressing neurons can also express Nts, which have been shown to act on GABAergic transmission in the CeA. Other neuropeptides, like SST, PKCδ, and substance P, may also regulate alcohol effects on the microcircuits in the CeA. The interplay between these neuropeptides and neuromodulatory systems with the endocannabinoid system and neuroinflammatory signaling may also alter the alcohol effect on neurotransmission in the CeA and alcohol-directed behaviors.

Overall, there are still many unknowns about the circuitry of the CeA and the role of specific various neuronal and non-neuronal cells and peptide systems in alcohol-related behaviors. Continued studies on intra-CeA microcircuitry as well as connectivity between the CeA and other brain regions may lead to novel therapeutic methods for treating AUD.

## Author Contributions

MM researched and reviewed the cited articles and wrote the manuscript. YS worked with MM to make conclusions based on existing literature, create the figures, and edited the manuscript. Both authors contributed to the article and approved the submitted version.

## Conflict of Interest

The authors declare that the research was conducted in the absence of any commercial or financial relationships that could be construed as a potential conflict of interest.

## Publisher’s Note

All claims expressed in this article are solely those of the authors and do not necessarily represent those of their affiliated organizations, or those of the publisher, the editors and the reviewers. Any product that may be evaluated in this article, or claim that may be made by its manufacturer, is not guaranteed or endorsed by the publisher.
